# Enhanced gastrointestinal stability and therapeutic efficacy of biofilm self-coated *Bacillus amyloliquefaciens* C-1 probiotic in ulcerative colitis

**DOI:** 10.1038/s41538-026-00889-2

**Published:** 2026-05-19

**Authors:** Jiayu Feng, Congyu Zhang, Yunyang Lu, Jiale Qin, Wanghong Su, Rui Dong, Huijuan Xi, Haoya Ma, Jia Lv, Yue Cheng, Haifeng Tang, Bei Han

**Affiliations:** 1 School of Public Health, Health Science Center, Xi’an Jiaotong University, Xi’an, Shaanxi China; 2 Department of Chinese Materia Medica and Natural Medicines, School of Pharmacy, Air Force Medical University, Xi’an, Shaanxi China; 3 Key Laboratory for Disease Prevention and Control and Health Promotion of Shaanxi Province, Xi’an, Shaanxi China

**Keywords:** Gastroenterology, Microbiology

## Abstract

Given the rising global incidence of inflammatory bowel disease (IBD) and limited treatment options, probiotic efficacy is hindered by poor gastrointestinal survival. This study developed a self-coating biofilm technology to encapsulate the probiotic *Bacillus amyloliquefaciens* C-1 and yield cC-1. Biofilm-modified cC-1 exhibited a more negative zeta potential (-22.53 mV) compared to uncoated C-1 (-19.60 mV), representing a decrease of 2.93 mV decreased zeta potential, and it exhibited orders of magnitude higher survival than uncoated cells under simulated gastric acid (80.51% *vs*. 0.33%) and bile salt (84.32% *vs*. 1.64%). In DSS-induced colitis mice, oral administration of 10⁹ CFU cC-1 for 14 days significantly alleviated symptoms, reduced colon shortening and restored mucosal integrity. Mechanistically, cC-1 effectively downregulated pro-inflammatory cytokines (TNF-*α*, IL-1*β*, IL-6 and IFN-*γ*) in colon tissues; reshaped the diversity of gut microbiota by enriching beneficial *Bacteroides*; restored linoleic acid and glycerophospholipid metabolism, and suppressed expression of NF-*κ*B signaling and cell adhesion molecule. Transcriptomic analysis confirmed that cC-1 reinstated host-microbe metabolic interactions with suppressed inflammatory pathways. This biofilm self-coating strategy substantially enhances probiotic gastrointestinal tolerance and exerts therapeutic effects through a multi-targeted mechanism (anti-inflammation, barrier repair, microbiota modulation, and metabolic reprogramming), offering a scalable and promising formulation for nutritional intervention in IBD.

## Introduction

The human intestinal microbiota plays a pivotal role in the host’s innate and adaptive immunity, metabolism, and overall health^1,2^. However, factors including lifestyle changes, dietary habits, infections and aging, disrupt this ecosystem^3^. Accumulating evidence links gut dysbiosis to various pathologies such as inflammatory bowel disease (IBD)^2^, obesity and psychiatric disorders^4,5^. Probiotic therapy has emerged as a pivotal strategy for restoring gut microbial homeostasis and alleviating aforementioned disorders. Despite its advantages, probiotic therapy faces challenges in clinical applications due to instability^6^. The complex and harsh gastrointestinal environment significantly impairs probiotic viability, leading to inadequate intestinal delivery, poor colonization, and consequently diminished bioavailability and therapeutic efficacy, ultimately limiting their probiotic effects^7^.

To address these limitations, multiple delivery systems have been developed for probiotic modification. Current strategies include physical encapsulation, microencapsulation, hydrogel-based approaches, and co-administration with prebiotics, among which biofilm-based methodologies have garnered considerable attention^8,9^. In natural environments, microorganisms are frequently embedded within self-synthesized extracellular polymeric matrices, a complex structure known as a biofilm, which is composed primarily of exopolysaccharides (EPS), proteins and other biomolecules^10^. Characterized by a hydrophobic interface, the biofilm layer is largely impermeable to aqueous liquids and organic solvents^11^. It facilitates bacterial adhesion to surfaces, prevents displacement by fluid flow, enhances survival under extreme conditions, and confers protection against host immune systems and antibiotics by acting as a penetration barrier^12,13^.

The existence of biofilms represents a double-edged sword. For pathogens, biofilm formation assists in evading host immune responses and is associated with the development of antibiotic resistance^14^. For probiotics, the protective properties of biofilms offer a novel strategy for probiotic delivery. Biofilms confer enhanced environmental resistance and improved adhesion capabilities to host tissues, thereby enabling probiotics delivered in biofilm form to exhibit superior survival rates, prolonged intestinal retention time, and enhanced therapeutic efficacy^15,16^.

*Bacillus* spp., including *Bacillus amyloliquefaciens*, possess the ability to form biofilms through complex regulatory networks involving quorum-sensing, two-component systems, and environmental signals, indicating potential for biofilm-based modification^17^. *B. amyloliquefaciens* is recognized as one of the bacterial species possessing Qualified Presumption of Safety (QPS) status by the European Food Safety Authority (EFSA)^18^. Strains in this genus, following rigorous safety and probiotic assessments, can be employed as commercial probiotic strains in human and animal food products^19^. Furthermore, both primary and secondary metabolites of *B. amyloliquefaciens*, such as EPS and lipopeptides, possess significant functional value, demonstrating a range of activities including antimicrobial, antiviral, anti-inflammatory, immunomodulatory, and antioxidant effects^20,21^.

*B. amyloliquefaciens* C-1, is a patented strain independently isolated by our laboratory. Previous studies have confirmed its excellent probiotic properties and high safety profile^22^. The developed postbiotic formulations have demonstrated considerable potential for the prevention and treatment of ulcerative colitis (UC) in zebrafish models^22^. Animal experiments have further verified that the whole fermentation broth of C-1 can effectively prevent and treat diarrhea in newborn calves. Moreover, it promotes growth in growth retarded calves by regulating growth hormone levels, improving intestinal and rumen development, and enhancing intestinal immunity^23^. These therapeutic effects suggest that the strain may offer valuable insights for IBD management.

IBD, including UC and Crohn’s disease (CD), is a chronic inflammatory disorder primarily affecting the colonic mucosa and submucosa. Patients with IBD commonly present with abdominal pain, diarrhea, rectal bleeding, anemia, weight loss and fatigue, which significantly compromise quality of life^24^. According to the Global Burden of Disease (GBD) database, the non-fatal health burden of IBD has continued to escalate^25^. Among digestive system diseases, IBD-related Years Lived with Disability (YLDs) rose from the fifth place in 1990 to fourth in 2021, surpassed only by upper digestive disorders, hernias, and cirrhosis^26^. Current IBD treatments predominantly rely on immunosuppression, with 5-aminosalicylic acid (5-ASA), corticosteroids, and TNF-*α* antibodies as first-line therapies^27,28^. However, these approaches lack curative efficacy and are limited by low bioavailability in inflamed intestinal regions, leading to suboptimal outcomes or severe side effects^29^. Additionally, broad-spectrum immunosuppressants such as steroids increase risks of infections and malignancies^30^. Given IBD’s association with microbial dysbiosis, probiotic therapy has emerged as a promising alternative choice. Probiotics modulate host immunity, enhance intestinal barrier integrity, regulate gut microbiota composition, and restore microbial balance to alleviate symptoms^31,32^.

Despite demonstrating multiple benefits, the application of probiotics in IBD treatment continues to face challenges of low bioavailability and instability, a finding corroborated by our preliminary research^22,23^. Although *B. amyloliquefaciens* C-1 possesses numerous advantages including documented anti-inflammatory properties, its efficacy as an oral probiotic formulation is compromised by pH stress encountered in gastrointestinal tract. A critical knowledge gap exists regarding whether harnessing the inherent biofilm-forming capacity of probiotic strains could serve as a natural, chemical-free strategy to enhance gastrointestinal survival while preserving or augmenting therapeutic efficacy. Inspired by biofilm-based modification strategies, and supported by our prior findings of EPS production and robust biofilm-forming capacity in C-1^33^, we hypothesized that inducing biofilm self-coating on *B. amyloliquefaciens* C-1 would enhance gastrointestinal stability and consequently improve therapeutic outcomes in ulcerative colitis. To verify it, we developed a biofilm self-coated formulation (cC-1), evaluated its gastrointestinal survival in vitro, then investigated its therapeutic efficacy and mechanisms in a DSS-induced colitis model through integrated histopathological, microbiological, metabolomic, and transcriptomic analyses. This self-coating technology is scalable, cost-effective, and free of synthetic chemicals, offering a promising platform for next-generation probiotic products. The current study evaluates this approach in a preclinical model, providing foundational evidence for its potential application in IBD and other gut-related disorders.

## Results

### Preparation and characterization of biofilm self-coated cC-1

**Fig. 1:**
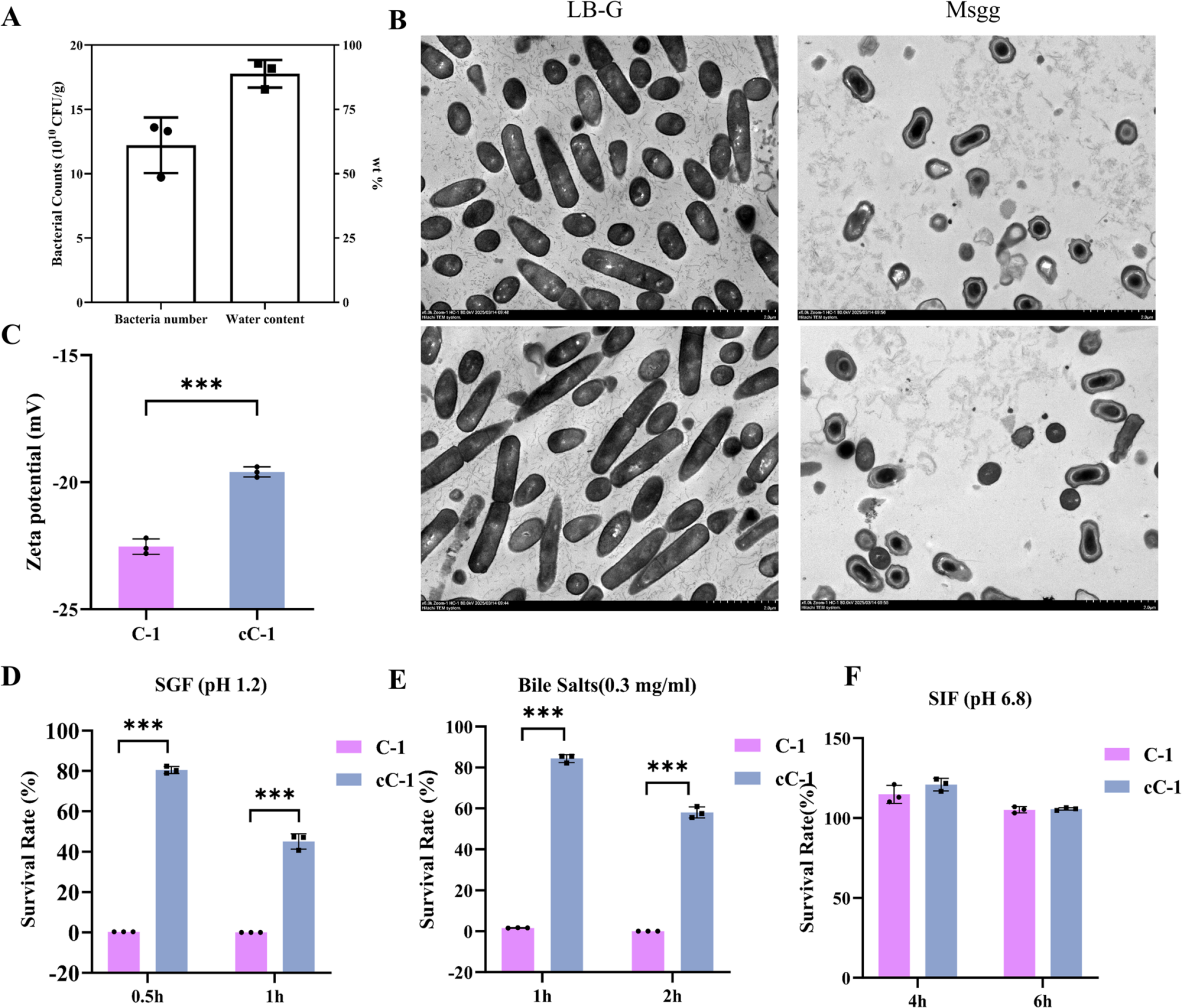
Bacteria quantification and biofilm observation (*n* = 3). **A** Contents of water loading and bacteria in the biofilms. **B** TEM images of *B. amyloliquefaciens* C-1 in LB-G and Msgg medium (right panel, cC-1). Scale bar = 2.0 μm. **C** Zeta potential characterization. **D** Equal amounts of C-1 (circle) and cC-1 (square) were separately exposed to SGF (pH 1.2, suppled with 3.2 g/L pepsin). **E** Equal amounts of C-1 (circle) and cC-1 (square) were separately exposed to bile salts (0.3 mg/mL). (**F**) Equal amounts of C-1 (circle) and cC-1 (square) were separately exposed to SIF (pH 6.8, suppled with 10 g/L trypsin). ***P < 0.001, Student’s *t* test.

Glucose significantly enhances EPS production by *B. amyloliquefaciens* C-1. Therefore, 1% glucose was added to the culture medium to boost biofilm formation, using MSgg medium to stimulate mature biofilm production. Rigorous quantification of cC-1 biomass was conducted to standardize bacterial quantification for subsequent experiments (Fig. 1A). cC-1 biomass was rigorously quantified. Results revealed a concentration of 12.2×10^10^ CFU/g of cC-1 biomass, with a moisture content of 88.84% (wet weight basis), which is characteristic of hydrated biofilm matrices.

TEM analysis confirmed the formation of a dense, encapsulating biofilm layer on cC-1 surfaces compared to the parental C-1 strain (Fig. 1B), aligning with our experimental hypothesis. The zeta potential measurements revealed a significant decrease of 2.93 ± 0.09 mV in biofilm-modified cC-1 compared to the C-1 control (-22.53 *vs*. -19.60, *P* < 0.001) (Fig. 1C). This reduction in surface negative charge indicates altered physicochemical properties of the bacterial surface following biofilm formation.

### The enhanced stress resistance of cC-1

The gastrointestinal (GI) environment presents significant challenges to probiotic viability due to its harsh physicochemical conditions, which impair bacterial delivery, colonization, and therapeutic efficacy by reducing bioavailability^5^. To evaluate stress resistance improvements post-modification, in vitro simulated GI assays were conducted. In SGF, the C-1 group exhibited severely compromised survival after 0.5 h of exposure (0.33%), whereas the cC-1 group maintained a significantly higher survival rate (80.51%, *P* < 0.001). After 1 h of exposure, the survival advantage of cC-1 was even more pronounced (45.08% vs. 0.01%, *P* < 0.001) (Fig. 1D). In bile salt solution, cC-1 demonstrated superior survival after 1 h (84.32% vs. 1.64%, *P* < 0.001) and after 2 h of exposure (58.04% vs. 0.004%, *P* < 0.001) (Fig. 1E). Notably, no significant difference was observed between C-1 and cC-1 groups in SIF after 4 h exposure (120.9% *vs*. 114.8%, *P* = 0.198) and 6 h (105.7% *vs*. 105.2%, *P* = 0.733), as both strains exhibited inherent resistance to intestinal pH (Fig. 1F). These findings collectively suggest that the self-coated modification enables cC-1 to better withstand GI stressors, thereby enhancing its potential for intestinal colonization and persistence.

### cC-1 intervention alleviates DSS-induced UC symptoms in mice

**Fig. 2:**
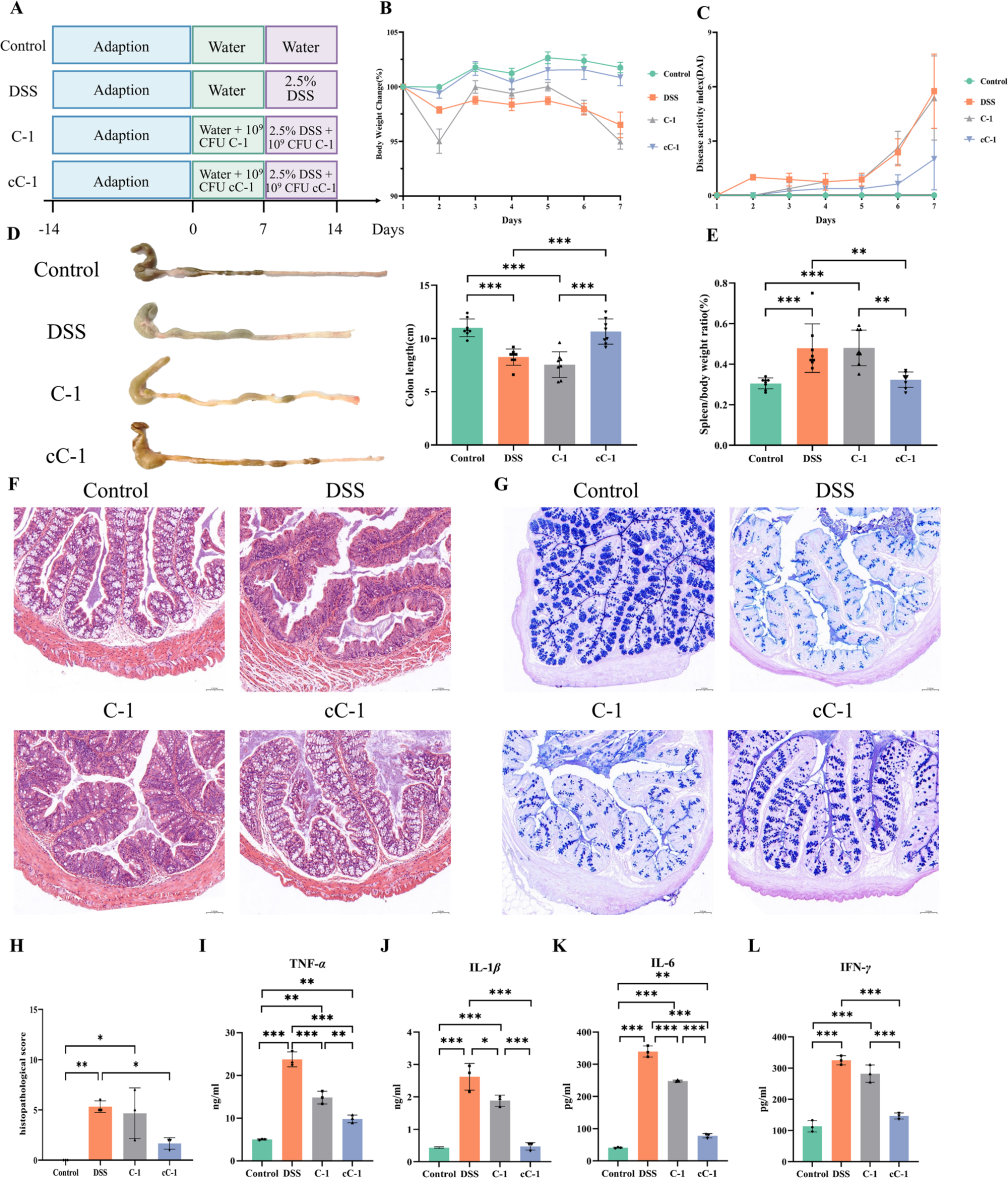
cC-1treatment alleviated DSS-induced ulcerative colitis symptoms in mice. **A** Diagram of the animal study design. **B** Body weight changes and **C** DAI scores of mice during DSS treatment (*n* = 8). **D** Representative photographs of the colon and statistics of colon length in each group. **E** The spleen index in mice from different groups. **F** Typical images of H&E staining of mice colon tissue (15**×**). **G** Typical images of AB-PAS staining of mice colon tissue (15**×**). **H** Histopathological score of H&E images. **I**–**K** The level of TNF-*α*, IL-1*β*, IL-6 and IFN-*γ* in the colon (*n* = 3). *P < 0.05, **P < 0.01, ***P < 0.001, One-way ANOVA.

A model of DSS-induced colitis was established to evaluate the protective effects of cC-1, and the specific experimental procedure is illustrated in Fig. 2A. During the DSS intervention, body weight and fecal status of the mice were recorded daily, and no mortality was observed throughout the study.

For the degrees of DAI score recorded during the whole experiment, the DSS-treated mice exhibited a dramatic increase over time, accompanied by significant weight loss, diarrhea, and hematochezia; Although the C-1 group showed a more gradual DAI score increase than the DSS group, it eventually developed comparable severity of weight loss, diarrhea, and hematochezia (DSS *vs*. C-1: 5.75 *vs*. 5.38, *P* = 0.970). In contrast, the cC-1 treatment group demonstrated marked improvements in weight maintenance, diarrhea reduction, and rare hematochezia occurrence compared to the DSS model group (DSS *vs*. cC-1: 5.75 *vs*. 2.00, *P* = 0.002). Furthermore, cC-1 treatment resulted in less pronounced weight loss and a more attenuated DAI score progression than C-1 treatment (cC-1 *vs*. C-1: 2.00 *vs*. 5.38, *P* = 0.004) (Fig. 2B, C and Figure S1).

Colon length served as an indicator of colitis severity. The DSS group showed significant colon shortening compared to controls (8.25 cm *vs*. 10.99 cm, *P* < 0.001), confirming successful UC model establishment. While C-1 administration also resulted in substantial colon shortening versus controls (7.54 cm *vs*. 10.99 cm, *P* < 0.001), suggesting limited therapeutic efficacy of oral C-1, the cC-1 group maintained normal colon length (10.65 cm *vs*. 10.99 cm, *P* = 0.910) and significantly prevented DSS-induced shortening (10.65 cm *vs*. 8.25 cm, *P* < 0.001) (Fig. 2D). The spleen index, reflecting systemic inflammation levels. DSS treatment significantly increased this ratio versus controls (0.48% *vs*. 0.31%, *P* < 0.001), which C-1 failed to ameliorate (0.48% *vs*. 0.31%, *P* < 0.001). Notably, cC-1 treatment normalized the ratio relative to both DSS model (0.32% *vs*. 0.48%, *P* = 0.002) and Control groups (0.32% *vs*. 0.31%, *P* = 0.963) (Fig. 2E).

Histopathological evaluation revealed distinct morphological differences. As shown in Fig. 2F, 2H and Figure S2, the normal group mice exhibited intact intestinal walls with well-defined crypt structures and orderly gland arrangement. Compared to the control group, DSS-treated UC mice displayed severe intestinal damage characterized by thinned intestinal walls, disrupted crypt architecture, irregular gland distribution, and inflammatory cell infiltration (*P* = 0.005). The intervention of cC-1 for DSS-treated UC mice showed the restored morphological structure to the normal group (*P* = 0.458), better than that of C-1 treatment (*P* = 0.011). The colonic mucus barrier, primarily secreted by goblet cells, constitutes the first line of intestinal defense. Impaired barrier function and increased intestinal permeability represent common complications of ulcerative colitis. AB-PAS staining demonstrated reduced mucus secretion in DSS-treated mice compared to healthy controls. cC-1 intervention effectively reversed this pathological alteration, exhibiting superior therapeutic outcomes over C-1 treatment (Fig. 2G; Figure S3).

For the inflammatory cytokines expression (TNF-*α*, IL-1*β*, IL-6 and IFN-*γ*) in colon issues, ELISA results demonstrated that DSS induction significantly elevated their levels versus controls (TNF-*α*: 23.76 ng/ml *vs*. 5.02 ng/ml, *P* < 0.001; IL-1*β*: 2.62 ng/ml *vs*. 0.43 ng/ml, *P* < 0.001; IL-6: 339.42 pg/ml *vs*. 40.76 pg/ml, *P* < 0.001; IFN-*γ*: 324.71 pg/ml *vs*. 113.22 pg/ml, *P* < 0.001). Compared to DSS group, C-1 moderately attenuated these elevations (TNF-*α*: 14.83 ng/ml *vs*. 23.76 ng/ml, *P* < 0.001; IL-1*β*: 1.88 ng/ml *vs*. 2.62 ng/ml, *P* = 0.019; IL-6: 247.77 pg/ml *vs*. 339.42 pg/ml, *P* < 0.001; IFN-*γ*: 282.01 pg/ml *vs*. 324.71 pg/ml, *P* = 0.091). As for cC-1 group, cC-1 not only markedly alleviated DSS-induced dysregulation of TNF-*α* (9.83 ng/ml *vs*. 23.76 ng/ml, *P* < 0.001), IL-1*β* (0.47 ng/ml *vs*. 2.62 ng/ml, *P* < 0.001), IL-6 secretion (77.91 pg/ml *vs*. 339.42 pg/ml, *P* < 0.001) and IFN-*γ* (146.38 pg/ml *vs*. 324.71 pg/ml, *P* < 0.001), but also demonstrated significantly stronger therapeutic effects compared to the C-1 group (TNF-*α*: 9.83 ng/ml *vs*. 14.83 ng/ml, *P* = 0.005; IL-1*β*: 0.47 ng/ml *vs*. 1.88 ng/ml, *P* < 0.001; IL-6: 77.91 pg/ml *vs*. 247.77 pg/ml, *P* < 0.001; IFN-*γ*: 146.38 pg/ml *vs*. 282.01 pg/ml, *P* < 0.001) (Fig. 2I-L). These findings collectively suggest that acid-resistant modification enhances bacterial survival in adverse gastrointestinal environments, thereby alleviating DSS-induced colitis symptoms.

### cC-1 treatment restored the intestinal microbiota of UC

**Fig. 3:**
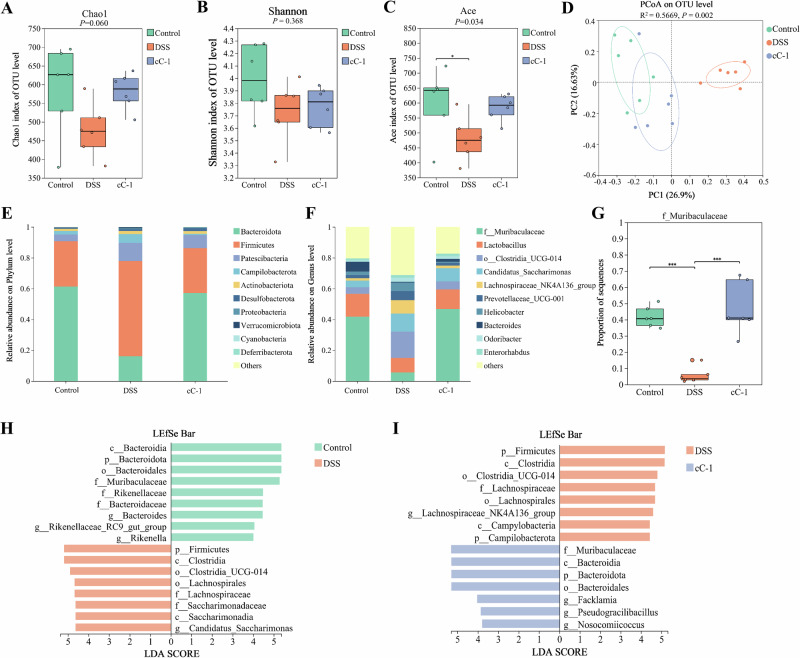
cC-1 modulated the composition of gut microbiota. **A**–**C** Alpha diversity index. **D** PCoA on OTU level. **E** The relative abundance of gut microbiota at phylum level. **F** The relative abundance of gut microbiota at genus level. **G** The relative abundance of *f_Muribaculaceae*. **H** LDA scores bar chart in Control *vs*. DSS. **I** LDA scores bar chart in DSS *vs*. cC-1. *P < 0.05, **P < 0.01, ***P < 0.001, One-way ANOVA with Tukey’s post-hoc test.

In the treatment of UC, restoration of the impaired gut microbiota represents a viable therapeutic approach for alleviating UC. Based on the aforementioned findings, we specifically investigated the restorative effects of biofilm-coated cC-1 on intestinal microbiota in DSS-induced UC. Supplied of cC-1 may restore the impaired gut microbiota of UC, as shown in Fig. 3A-C, significant differences were observed among the Control, DSS, and cC-1 groups in alpha diversity of ACE index (*P* = 0.034). Compared to controls, DSS-treated mice exhibited significantly reduced ACE index (479.21 *vs*. 602.97, *P* = 0.045), which shown lower richness. Following cC-1 intervention, compared to the DSS group, we observed numerical improvements in richness and diversity (Chao1: 581.74 *vs*. 477.47, *P* = 0.116; Shannon: 3.77 *vs*. 3.73, *P* = 0.942; Ace: 584.63 *vs*. 479.21, *P* = 0.093), with no statistical significance. *β*-diversity analysis using Bray-Curtis distance-based PCoA revealed distinct microbial community structures among the three groups (*R*² = 0.567, *P* = 0.002). PERMANOVA testing demonstrated significant separation between Control and DSS groups (*R*^2^ = 0.330, *P* = 0.005). Notably, the cC-1 group showed intermediate clustering that was not significantly different from Control (*R*^2^ = 0.162, *P* = 0.063) but remained significantly different from the DSS group (*R*^2^ = 0.269, *P* = 0.002), indicating that cC-1 treatment partially restored the gut microbial community structure toward that of healthy controls (Fig. 3D).

For the relative abundance of gut microbiota, Bacteroidota and Firmicutes remained the dominant phyla across all groups. Compared with the Control group, DSS treatment significantly reduced the abundance of Bacteroidota (16.15% *vs*. 61.38%, *P* < 0.001) while markedly increasing Firmicutes levels (61.73% *vs*. 29.39%, *P* < 0.001). Notably, cC-1 administration effectively reversed these DSS-induced alterations in Bacteroidota (57.20% *vs*. 16.15%, *P* < 0.001) and Firmicutes abundance (29.03% *vs*. 61.73%, *P* < 0.001), restoring their proportions to levels comparable with the Control group (Bacteroidota: 57.20% *vs*. 61.38%, *P* = 0.835; Firmicutes: 29.03% *vs*. 61.73%, *P* = 0.532) (Fig. 3E and Table S4). Compared to the control group, mice in the DSS group showed a significantly lower abundance of the family *Muribaculaceae* in intestinal contents (5.53% *vs*. 41.74%, *P* < 0.001). Administration of cC-1 restored its abundance to 46.79% (*vs*. 5.53% in DSS group, *P* < 0.001). At the genus level, cC-1 treatment also significantly increased the abundance of *Bacteroides* (belonging to *Bacteroidaceae*) from 1.23% in DSS group to 8.67% (*P* < 0.001), suggesting that both *Muribaculaceae* and *Bacteroides* may contribute to the therapeutic effects (Fig. 3F, G and Table S5).

LEfSe was employed to assess taxonomic hierarchies and microbial abundance variations across groups with LDA scores > 3. The results revealed distinct shifts in dominant microbial taxa among experimental groups. In Control group, the predominant taxa were phylum Bacteroidota and genus Bacteroides. In DSS group, phylum Fimicutes and genus Clostridia_Saccharimonas became dominant. In cC-1 group, compared with DSS group, phylum Bacteroidota became the prevailing taxa again. (Fig. 3H and I).

**Fig. 4:**
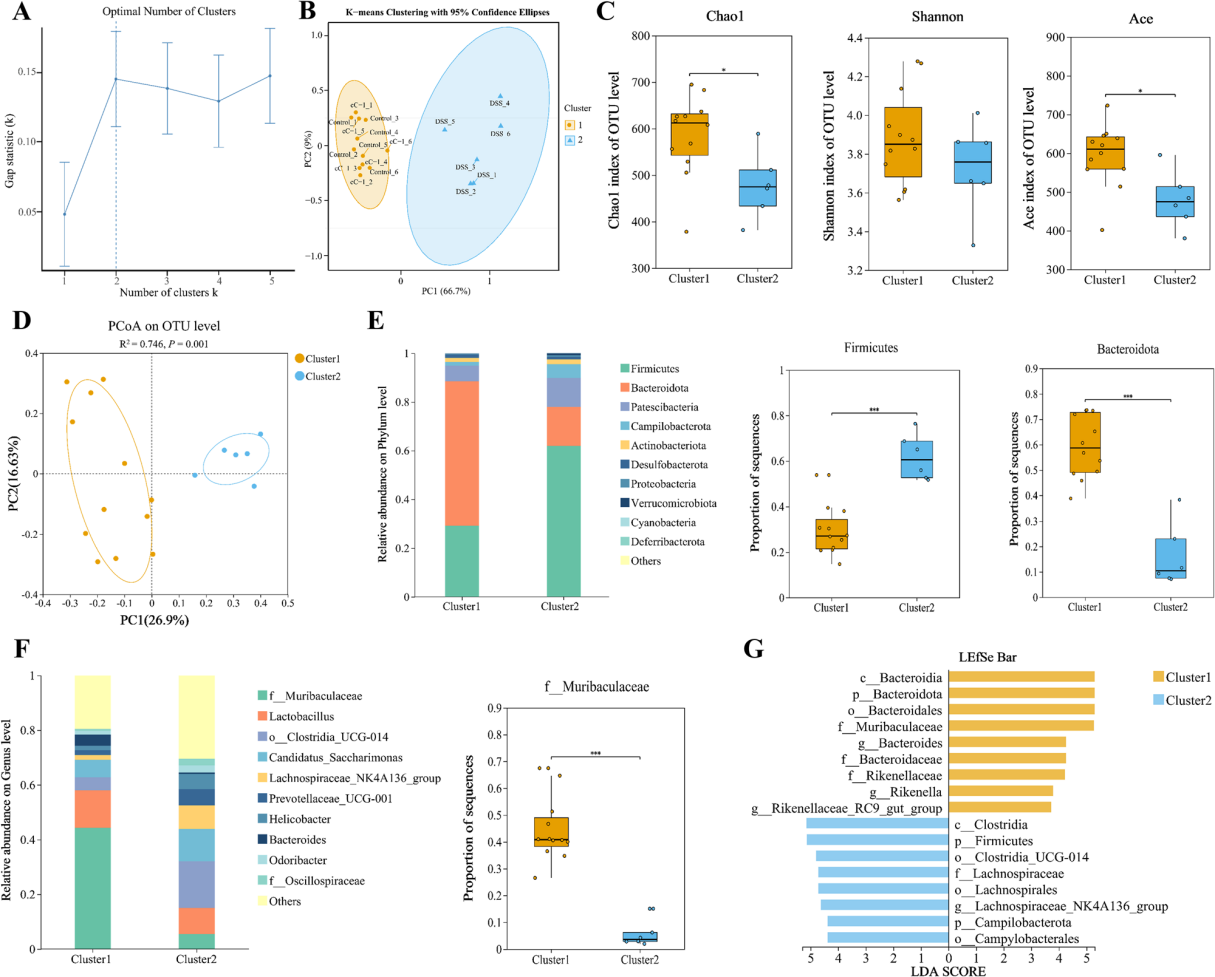
The different composition of gut microbiota among two clusters separated by K-means. **A** The optimal number of clusters based on the gap statistic. **B** K-means clustering plot by Bray-Curtis distance based on the optimal number clusters. **C** Alpha diversity index. **D** PCoA on OTU level. **E** The relative abundance of gut microbiota at the phylum level. **F** The relative abundance of gut microbiota at the genus level. **G** LDA scores bar chart among two clusters. **P < 0.01, ***P < 0.001.

To further characterize the microbial composition of the samples, k-means clustering was performed. The optimal cluster number (*k* = 2) was determined by gap statistical analysis, resulting in two distinct clusters (Fig. 4A): Cluster 1 containing 12 samples, all from the Control (*n* = 6) and cC-1 treatment groups (*n* = 6), and Cluster 2 containing all 6 samples from DSS-treated group (*n* = 6). This clustering pattern demonstrates a closer microbial similarity between the Control and cC-1 groups compared to the DSS group (Fig. 4B). Cluster 1 showed higher community richness than that of cluster 2 (sobs: 493.08 *vs*. 411.50, *P* = 0.034; Chao1: 585.64 *vs*. 477.47, *P* = 0.035; Ace: 593.80 *vs*. 479.21, *P* = 0.010). In contrast, no significant differences were observed in the community diversity indices (Shannon: 3.88 *vs*. 3.73, *P* = 0.223; Simpson: 0.05 *vs*. 0.06, *P* = 0.632) between the two clusters (Fig. 4C). PCoA revealed distinct microbial community structures (*R*² = 0.75, *P* = 0.001) and PERMANOVA testing demonstrated significant separation between two clusters (*R*^2^ = 0.76, *P* < 0.001) (Fig. 4D).

At the phylum level, Bacteroidota and Firmicutes remained the dominant phyla in the gut microbiota. Compared to cluster 1, as shown in Fig. 4E, cluster 2 treatment significantly reduced the abundance of Bacteroidota (16.15% *vs*. 59.29%, *P* < 0.001) while markedly increasing the relative abundance of Firmicutes (61.73% *vs*. 29.21%, *P* < 0.001). At the genus level, cluster 2 continued to exhibit a significant reduction in the abundance of family *Muribaculaceae* (5.53% *vs*. 44.27%, *P* < 0.001), which further suggests that this bacterium may play an important role in the disease process (Fig. 4F). LEfSe results revealed distinct shifts in dominant microbial taxa among clusters. The predominant taxa in cluster 1 were the phylum Bacteroidota and the genus *Bacteroides*, whereas cluster 2 was primarily dominated by the phylum Firmicutes. (Fig. 4G).

### Untargeted metabolomics analysis of intestinal contents

**Fig. 5:**
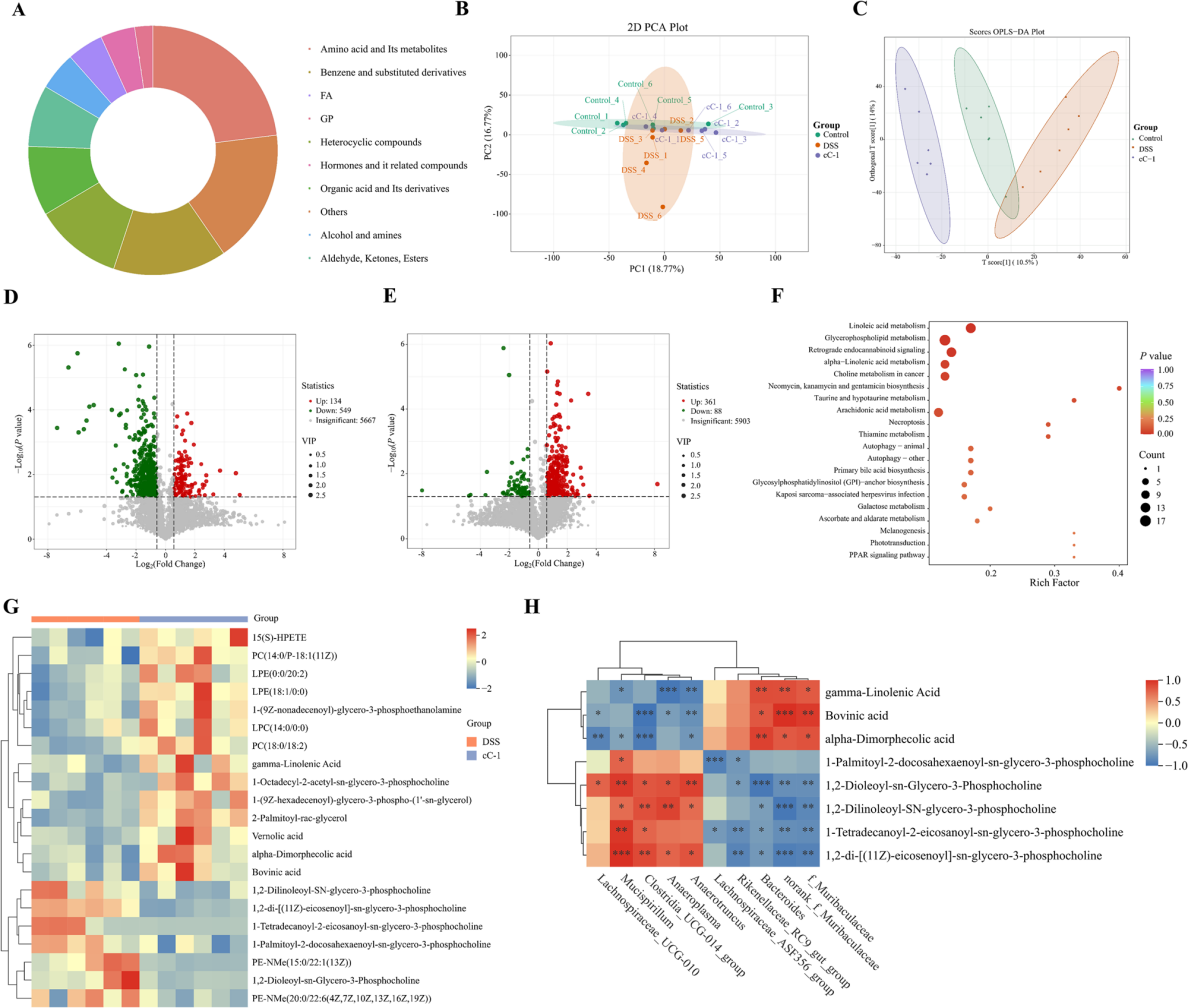
Effect of cC-1 on untargeted metabolomics of feces. **A** Pie chart showing the distribution of metabolite classes identified in feces across all samples, representing the actual metabolomic composition detected. **B** PCA among three groups (Control, DSS and cC-1). **C** OPLS-DA score plot among three groups. **D** Volcano map of differential metabolites in DSS *vs*. Control group. **E** Volcano map of differential metabolites in cC-1 *vs*. DSS group. **F** KEGG functional enrichment analysis in cC-1 *vs*. DSS comparison (pathway enrichment). **G** Metabolites expression heat map between DSS and cC-1 groups. **H** Spearman correlation map between differential metabolites and core bacterial genera. *P < 0.05, **P < 0.01, ***P < 0.001.

In this study, untargeted metabolomics was employed to analyze mouse feces, aiming to explore the intrinsic relationship between metabolites and pathological changes across different groups. Differential metabolites were screened using the criteria of FoldChange ≥ 1.5 and *P*_*adj*_ < 0.05. After data preprocessing, a total of 7,206 metabolites were identified in both positive and negative ion modes, spanning 25 major categories-including Amino acids and its Metabolites, Benzene and Substituted Derivatives, and Fatty Acids, among others (Table S6 and Fig. 5A). PCA revealed that the metabolite profiles of the Control and cC-1 groups were more similar to each other than to those of the DSS group (Fig. 5B).

To better eliminate irrelevant variances and screen for meaningful differential variables, OPLS-DA was performed on the three groups. As illustrated in Fig. 5C, the Control and cC-1 groups clustered more closely together compared to the DSS group. Relative to the Control group, the DSS group exhibited 683 differential metabolites, of which 134 were upregulated and 549 were downregulated (Fig. 5D). In the comparison between the cC-1 and DSS groups, 449 differential metabolites were identified, comprising 361 upregulated and 88 downregulated metabolites (Fig. 5E).

KEGG pathway enrichment analysis of these differential metabolites indicated that, compared to the DSS model group, the metabolites altered after cC-1 treatment were primarily associated with Linoleic acid metabolism, Glycerophospholipid metabolism, and Arachidonic acid metabolism, among other pathways (Fig. 5F). Furthermore, 21 core differential metabolites were screened and identified between the DSS and cC-1 groups, demonstrating that cC-1 treatment notably ameliorated the metabolic disturbances induced by DSS administration (Fig. 5G).

To further investigate the relationship between gut microbiota and metabolites, Spearman correlation analysis was conducted between the differential metabolites identified via untargeted metabolomics and the core genera screened by 16S rRNA sequencing, with correlation coefficient absolute value threshold > 0.8 and *P*_adj_ <0.05 applied to filter significant associations. The results demonstrated that cC-1-enriched genera like *Bacteroides*, *Lachnospiraceae_UCG-010*, *Rikenellaceae_RC9_gut_group* and *f_Muribaculaceae* showed significant correlations with differential metabolites, suggesting a potential role for these taxa in mediating the therapeutic effects of cC-1. (Fig. 5H). This suggests that cC-1 exerts its therapeutic effect not merely by altering microbial composition, but by restoring critical microbiota-host metabolic crosstalk, thereby promoting a systemic resolution of inflammation and tissue damage.

### Transcriptional profiles analysis of the intestinal tissues

To elucidate the molecular mechanisms underlying cC-1’s therapeutic effects, we performed RNA-seq transcriptomic analysis on colon tissue samples to compare gene expression profiles among Control, DSS, and cC-1 treatment groups. Differentially expressed genes (DEGs) were identified using stringent criteria (|log_2_ FC | ≥1, *P*_adj_ < 0.05).

**Fig. 6:**
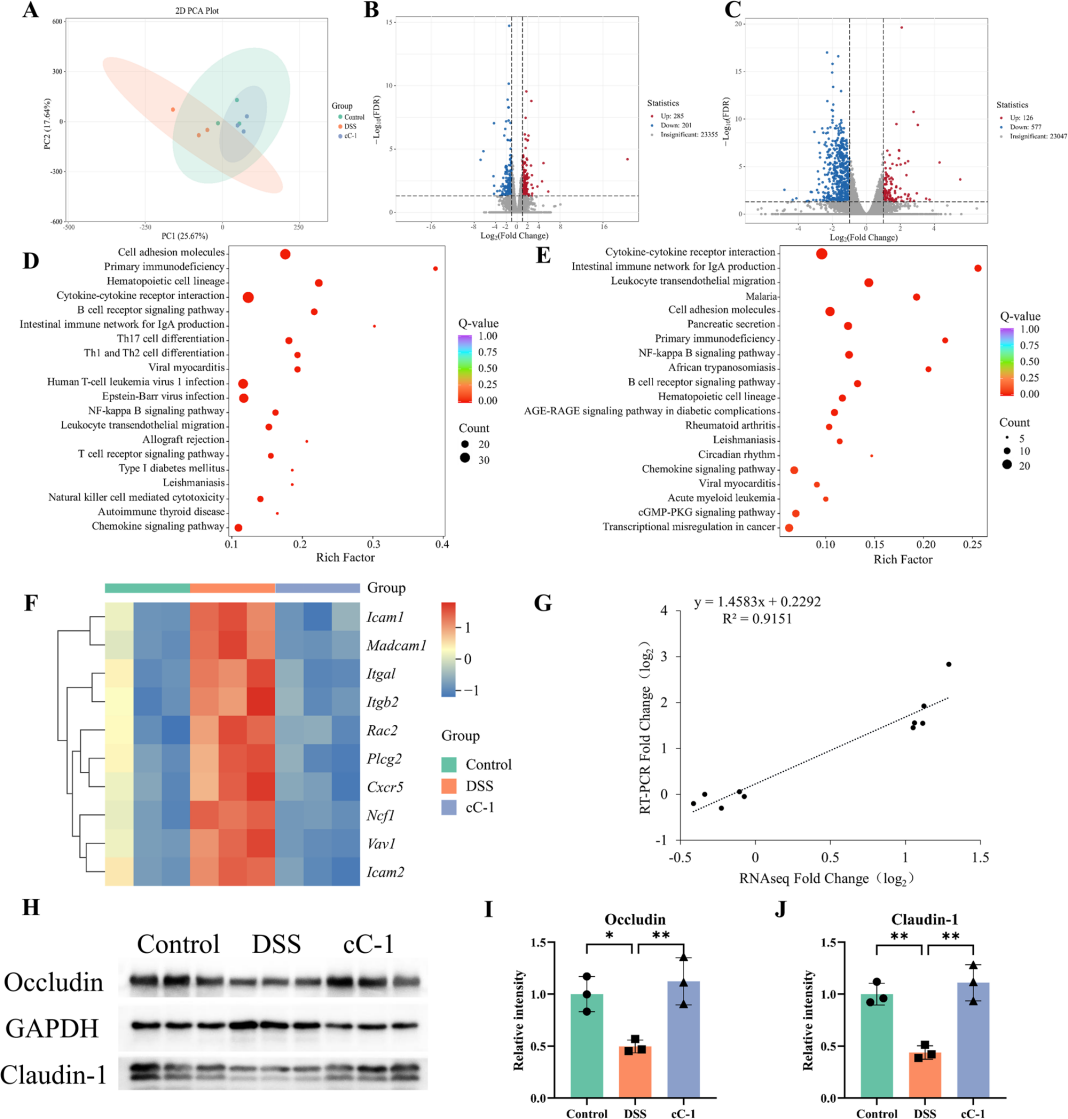
Effect of cC-1 on transcriptional profiles. **A** PCA among three groups (Control, DSS and cC-1). **B** Volcano map of differential metabolites in DSS *vs*. Control group. **C** Volcano map of differential metabolites in cC-1 *vs*. DSS group. **D** KEGG functional enrichment analysis in DSS *vs*. Control comparison (pathway enrichment). **E** KEGG functional enrichment analysis in cC-1 *vs*. DSS comparison (pathway enrichment). **F** Gene expression heat map among different groups. **G** Correlation between Q-RT-PCR and transcriptome data. **H** Typical images of western blotting. **I**–**J** Relative expression of Occludin and Claudin-1 (*n* = 3). *P < 0.05, **P < 0.01, One-way ANOVA.

PCA demonstrated distinct clustering patterns, revealing greater similarity between cC-1-treated and Control groups compared to DSS-induced samples (Fig. 6A). Comparative analysis identified 486 significant DEGs in DSS-treated mice versus controls, comprising 285 upregulated and 201 downregulated genes (Fig. 6B). Notably, cC-1 administration modulated 603 DEGs relative to the DSS group, with 126 upregulated and 577 downregulated transcripts (Fig. 6C). Pathway enrichment analysis demonstrated that DEGs in the DSS group were predominantly associated with cell adhesion molecules, NF-*κ*B signaling pathway and leukocyte transendothelial migration when compared to the Control group. Notably, when comparing cC-1-treated mice to the DSS model group, the differentially expressed genes were significantly enriched in pathways similar to those altered in the DSS *vs*. Control comparison, including cell adhesion molecules and NF-*κ*B signaling pathway, indicating that cC-1 treatment reverses DSS-induced transcriptional changes (Fig. 6D, E).

Of particular significance, chemokine-related genes (*Rac2*, *Plcg2*, *Ncf1*, *Cxcr5* and *Vav1*) and cell adhension related genes (*Icam1*, *Icam2*, *Itgal*, *Itgb2* and *Madcam1*) exhibited marked upregulation in the DSS model group, which was subsequently attenuated by cC-1 treatment (Fig. 6F). To validate the transcriptomics results, five genes were randomly selected for Q-RT-PCR analysis, the outcomes of which were consistent with the transcriptomic data (Fig. 6G and Figure S4). We also examined the expression of tight junction proteins in colonic tissues (Fig. 6H–J and Figure S5). Western blotting revealed that compared with the normal group, the expression of both Occludin (*P* = 0.024) and Claudin-1 (*P* = 0.003) was significantly decreased in the UC group. Administration of cC-1 notably attenuated this DSS-induced reduction in the expression of Occludin (*P* = 0.009) and Claudin-1 (*P* = 0.001). These findings collectively suggest that cC-1 may exert its effects by reducing local tissue inflammation, improving intestinal permeability, and promoting the repair of damaged intestinal mucosa, which aligns with the observations from histopathological analysis and ELISA assays.

## Discussion

This study investigated the feasibility of biofilm-coated probiotics and preliminarily examined their therapeutic efficacy and mechanisms in a DSS-induced murine model of ulcerative colitis. Our findings indicate that cC-1 significantly enhances gastrointestinal survival, ameliorates colitis symptoms, restores intestinal barrier integrity, and modulates gut microbiota composition, highlighting its promise as a novel probiotic-based intervention for UC. Probiotic therapy has attracted considerable interest as a strategy for managing UC, owing to its ability to modulate immune responses, reinforce epithelial barriers, and restore microbial homeostasis^34–36^. However, the clinical translation of probiotics is often hampered by their vulnerability to harsh gastrointestinal conditions, resulting in poor viability and limited therapeutic efficacy^5^. To address this, we focused on *B. amyloliquefaciens* C-1, a strain previously documented for its anti-inflammatory properties and postbiotic potential in UC models^23,33^. Leveraging its inherent capacity for exopolysaccharide production and biofilm formation, we developed a biofilm-based coating strategy aimed at enhancing environmental resilience and gastrointestinal persistence.

The protective role of biofilms is well-established in microbial ecology, where they serve as physical barriers against environmental stressors, promote adhesion to host tissues, and facilitate community-level interactions^8–13^. In the present study, TEM imaging and zeta potential analysis confirmed the successful formation of a dense, cohesive biofilm layer on cC-1. Such changes may promote adhesion to the intestinal mucosa and modulate interactions with host immune cells. This modification conferred remarkable resistance to simulated gastric fluid and bile salts, thereby addressing a key limitation in oral probiotic delivery. The survival rate of cC-1 increased by 243-fold in SGF (80.51% *vs*. 0.33%) and by 51-fold in 0.3% bile salts (84.32% *vs*. 1.64%). Notably, both C‑1 and cC‑1 exhibited an apparent increase in viable counts after exposure to SIF. We speculate that this phenomenon reflects an adaptive response of *B. amyloliquefaciens* C‑1 to the specific environmental stimulation of the intestinal fluid, which may enhance the rates of cell division and spore formation as a strategy to counteract environmental stress. Such an adaptive response likely contributes to improved transit tolerance within the intestine and may facilitate better colonization. This protective effect was associated with an increased biofilm thickness of 2.93 ± 0.09 μm and enhanced surface stability, as indicated by a shift in zeta potential from −19.6 mV to −22.53 mV. These in vitro findings provide a mechanistic basis for the improved in vivo performance of cC-1.

In the DSS-induced murine model of UC, administration of cC-1 significantly alleviated disease severity. The reduction in the DAI score, improvement in colonic morphology, and decreased levels of pro-inflammatory cytokines (TNF-*α*, IL-1*β*, IL-6, and IFN-*γ*) collectively highlight the anti-inflammatory and tissue-protective properties of cC-1. Histopathological examination further demonstrated the in vivo anti-inflammatory effect of cC-1. AB-PAS staining revealed that cC-1 treatment mitigated the DSS-induced reduction in mucin secretion by colonic goblet cells. In combination with Western blotting analysis of tight junction protein expression (Occludin and Claudin1), these findings indicate that cC-1 administration ameliorated DSS-induced colonic barrier damage - a critical factor in the recovery process of UC patients. Effective recovery in UC not only requires attenuation of intestinal inflammation and reduction of associated inflammatory immune cells, but also relies on epithelial cell proliferation and regeneration to heal mucosal ulcers^37^. To further elucidate the molecular mechanisms underlying cC-1’s efficacy, we performed transcriptomic analysis of colonic tissues. Pathway enrichment highlighted significant modulation of the NF-*κ*B signaling pathway and cell adhesion molecules, both centrally implicated in UC immunopathology^38–41^. Furthermore, cC-1 treatment downregulated the overexpression of *Plcg2* (FC = −0.347, *P* < 0.001), *Vav1* (FC = −0.360, *P* < 0.001), *Ncf1* (FC = −0.355, *P* < 0.001), and *Rac2* (FC = −0.388, *P* < 0.001). *Plcg2* is an upstream initiator of *Vav1*, and the protein encoded by *Ncf1* is a key component of the NADPH oxidase complex. Notably, *Vav1*, a crucial adaptor protein downstream of *Plcg2*, activates Rac family proteins, which in turn can activate NADPH oxidase to generate ROS, thereby exacerbating tissue damage. The simultaneous downregulation of these five genes by cC-1 suggests that its therapeutic effect may also involve the inhibition of intracellular oxidative stress-related signaling pathways.

Extensive research has established the critical involvement of gut microbiota in UC pathogenesis^42^. The intestinal microbiome plays vital roles in maintaining host health through its functions in nutrition metabolism, immune system development, and host defense mechanisms. Notably, dysbiosis of commensal microbiota can both trigger excessive inflammatory responses and result from impaired innate/adaptive immunity^43^. Analysis revealed that *Muribaculaceae* may serve as a key mediator of the therapeutic effects of cC-1. Its abundance was significantly reduced in the UC model group, while cC-1 treatment markedly restored its levels. This restoration showed a positive correlation with changes in differential metabolites identified in the metabolomic analysis. As a member of the order Bacteroidales, family *Muribaculaceae* has been reported to be markedly decreased in patients with IBD-a finding consistent with the present results^44^. This restitution correlated with improved metabolic profiles, as indicated by untargeted metabolomics, suggesting that cC-1 exerts its therapeutic effects partly through microbial rebalancing and associated metabolic reprogramming.

In addition, our metabolomic analysis revealed that cC-1 intervention significantly modulated several key metabolic pathways, including linoleic acid, glycerophospholipid, and arachidonic acid metabolism, which are closely associated with inflammatory responses and membrane integrity. Compared to the model group, the cC-1 group exhibited a coordinated metabolic reprogramming pattern: multiple lysophospholipids (e.g., LPC (14:0/0:0) and LPE (18:1/0:0)), free fatty acids (e.g., *γ*-linolenic acid), and 15(S)-HPETE, a 15-lipoxygenase (15-LOX) metabolite of arachidonic acid, were significantly elevated. The increase in precursors such as *γ*-linolenic acid, an n-6 polyunsaturated fatty acid, suggests a potential shift in metabolism toward the synthesis of the anti‑inflammatory mediator dibomo‑*γ*‑linolenic acid^45^. More importantly, the elevation of 15(S)-HPETE, a key intermediate in the pro‑resolving pathway, strongly indicates that the treatment may redirect arachidonic acid metabolism from the classical pro‑inflammatory prostaglandin and leukotriene synthesis toward the 15‑LOX‑mediated production of pro‑resolving mediators (e.g. lipoxins, resolvins). 15(S)-HPETE serves as a precursor for lipoxin A4 (LXA4) and regurgin D1 (RvD1). We hypothesize that cC-1 redirects pro-inflammatory arachidonic acid metabolism from the PGE2/LTB4 axis to the pro-resolving LX/Rv axis by activating the 15-LOX pathway, thereby explaining the downregulation of inflammatory mediators such as TNF-*α*. Concurrently, the restoration of various intact phospholipid species suggests improved integrity of the intestinal epithelial cell membrane.

Correlation analysis with 16S data indicated that in the treatment group, γ-linolenic acid and bovinic acid (a conjugated linoleic acid) showed significant positive correlations with the abundance of specific gut bacteria, such as *Muribaculaceae* and *Bacteroides*. These associations suggest that cC-1 may alleviate colitis symptoms by promoting beneficial bacteria such as *Muribaculaceae* and *Bacteroides*, thereby creating an intestinal microenvironment favorable for host synthesis of pro-resolving mediators and restoration of cellular membrane integrity.

The cC-1 exhibited superior therapeutic outcomes, underscoring the importance of biofilm modification in enhancing probiotic functionality. This aligns with emerging approaches that employ material-assisted probiotic delivery to improve targeting and persistence. However, several limitations warrant consideration. First, while cC-1 was comprehensively evaluated, the parental C-1 strain did not undergo parallel mechanistic profiling, limiting direct comparative interpretation of biofilm-specific effects. Second, only a single dose (10^9^ CFU/day) was tested; no dose-response curve was established, making it impossible to determine the minimum effective dose and optimal therapeutic window. Although transcriptomic data implicated barrier-related pathways, tight junction gene expression did not show significant differential expression, suggesting potential post-transcriptional regulation not captured in this study. Third, the use of a chemically induced murine model may not fully recapitulate the chronic, immune-mediated complexity of human UC. Fourth, the experimental design employed in this study represents a combined prophylactic and therapeutic intervention, as mice received probiotic treatment for 7 days before DSS exposure (prophylactic phase) and continued during the 7 days of DSS administration (therapeutic phase). While this design effectively demonstrates that cC‑1 can both prevent and ameliorate DSS‑induced colitis, it differs from the typical clinical scenario where patients seek treatment after symptoms have already developed.

This study presents a biofilm self-coated *B. amyloliquefaciens* C-1 (cC-1) suitable for experimental colitis, which combines enhanced resilience and therapeutic effect. As the next-generation probiotic platform, it is expected to enhance therapeutic effects, achieve targeted drug delivery, and prevent intestinal infections. Future studies should evaluate cC-1 in chronic UC models (e.g., *IL*-*10*^−^/^−^ mice) and larger animals for colonization persistence. The utilization of biofilm self-coating engenders regulatory advantages in comparison with microencapsulation, by virtue of the avoidance of synthetic materials (sodium alginate). The spore-forming capability of *B. amyloliquefaciens* may enhance shelf-life stability beyond *E. coli* Nissle 1917. While spontaneous biofilm formation is cost-effective for industrialization, batch consistency requires optimization of fermentation parameters (pH, dissolved oxygen, harvest timing).

## Methods

### Bacterial strains and growth conditions

*B. amyloliquefaciens* C-1 is a patented strain isolated by our laboratory (CCTCC NO: M2012177), which grows in Luria-Bertani-glucose (LB-G) medium (Beijing Land Bridge Technology Co., Ltd., CM1552, China) containing 10 g/L peptone, 5 g/L yeast extract, 10 g/L NaCl, and 10 g/L glucose, with pH 6.8–7.2. For solid medium preparation, 15 g/L agar was extra added. Cultures were incubated aerobically at 30 °C for 12-18 hours with shaking at 200 rpm in 50 mL medium within 250 mL flasks. Working cultures were initiated at OD₆₀₀ = 0.05 from overnight pre-cultures. All experiments used vegetative cells in mid-log phase (OD₆₀₀ = 0.6–0.8), confirmed by microscopy to be >99% vegetative cells with no detectable spores.

### Preparation and quantification of cC-1

Biofilm self-coated *B. amyloliquefaciens* C-1 (cC-1) was produced through the third-generation activated C-1 cells growing in Minimal salts glycerol glutamate (MSgg) induction medium (Shanghai Huzhen industrial Co., Ltd, 0130, China), containing 0.1 M MOPS (3-(N-morpholino) propanesulfonic acid), 2 mM MgCl₂, 50 µM MnCl₂, 1 µM ZnCl₂, 1 µM thiamine, 700 µM CaCl₂, and 100 µM FeCl₃, with 15 g/L agar powder for solid medium preparation. Activated C-1 (defined as cells from the third subculture after frozen stock, grown to mid-log phase with OD₆₀₀ = 0.6–0.8) was first inoculated into MSgg liquid medium and cultured overnight at 30 °C. Subsequently, 100 µL of the bacterial suspension was spread onto MSgg agar plates and incubated at 30 °C for 72 h to induce the formation of biofilm self-coated cC-1. For quantitative analysis, cC-1 biomass was carefully scraped from MSgg agar plates using a sterile cell scraper, collected into pre-weighed sterile tubes, and weighed. 10 mg of this biomass (wet weight) was resuspended in 1 mL PBS, subjected to serial dilution and plated onto LB-G agar. After overnight incubation at 30 °C, colony-forming units (CFU) were enumerated to establish the correlation between cC-1 mass and viable cell count.

### TEM observation of cC-1 cells

For ultrastructural observation of cC-1 cells, the method is improved accordingly^46^. Bacterial cells were washed twice with sterile PBS, centrifuged at 4 °C (5000 × g, 10 min) to remove supernatants, and fixed overnight in 2.5% glutaraldehyde (Sigma-Aldrich, G5882, USA) at 4 °C. The pelleted cells were rinsed three times with 0.2 M PBS buffer, followed by stepwise dehydration using a graded ethanol series (30%, 50%, 70%, 80%, 90%, and 100%; 15 min each). Dehydrated samples were embedded in epoxy resin (Embed-812, Electron Microscopy Sciences, 14120) within molds, polymerized at 60 °C for 48 h, and sectioned (70 nm thick) using an ultramicrotome (Leica UC7, Germany). Ultrathin sections were mounted on grids, stained with 2% uranyl acetate and lead citrate, and examined under a transmission electron microscope (TEM, Hitachi HT7800, Japan) in the Instrumental Analysis Center of Xi’an Jiaotong University.

### Biofilm water content and zeta potential analysis of cC-1

Water content and zeta potential measurements were performed to evaluate the hydration status and structural stability of the biofilm, as well as to characterize the surface charge properties and adhesion potential of the bacteria. The biofilm water content was determined by drying 50 mg bacterial biomass (wet weight) in a 65 °C oven until constant weight achieved, then moisture content was calculated. For zeta potential analysis, aliquots of C-1 and cC-1 bacterial suspensions were prepared in 1 mM KCl. Bacteria were washed twice and resuspended in 1 mM KCl (pH 7.0) to a final concentration of approximately 10^6^ cells/mL. Each sample was measured in triplicate with 20 runs per measurement at 25 °C. Measurements were performed using a Zetasizer Nano ZSE instrument (Malvern Zetasizer Nano ZSE, UK) in the Instrumental Analysis Center of Xi’an Jiaotong University.

### Stress resistance assessment of cC-1 in vitro

Simulated digestive fluids were prepared, including simulated gastric fluid (SGF, 2 g/L NaCl, 3.2 g/L pepsin, and 7 mL/L hydrochloric acid with pH 1.2) and simulated intestinal fluid (SIF, 6.8 g/L KH₂PO₄, 77 mL/L 0.2 M NaOH, and 10 g/L trypsin, adjusted to pH 6.8)^47^. A 0.3 mg/mL bile salt solution was prepared by dissolving 0.03 g porcine bile salts (Sigma-Aldrich, B8631) in 100 mL LB-G medium. A defined bacterial biomass (equivalent to 10⁸ CFU based on pre-determined CFU/mass correlation) was resuspended in 1 mL of SGF, SIF, or bile salt solution, separately, and incubated at 37 °C for specified durations (0.5 h and 1 h for SGF; 1 h and 2 h for bile salts; 4 h and 6 h for SIF). Subsequently, 50 μL aliquots were serially diluted and plated for colony counting to calculate survival rates. Survival rate was calculated as (CFU after treatment / initial CFU) × 100%. Initial CFU was determined by plating serial dilutions of the bacterial suspension immediately before exposure and the C-1 was used as control.

### Dextran sulfate sodium (DSS)-induced mouse model of colitis

Specific pathogen-free (SPF) male Balb/c mice (5-6 weeks old upon arrival, *n* = 32 total) were obtained from Laboratory Animal Center (LAC) of The Air Force Medical University and maintained on a standard diet. Housing conditions included a 12-h light/dark cycle, 50-60% relative humidity, 21–26 °C ambient temperature, with regular replacement of bedding and water. The animal experiments conducted in this study were performed in accordance with the International Guiding Principles for Biomedical Research Involving Animal and approved by the Ethics Committee of Xi’an Jiaotong University Health Sciences Center (No. 2021-014).

After a two-week acclimatization period (mice were thus approximately 7–8 weeks old at experiment initiation), mice were stratified by body weight, labeled, and randomly divided into four groups (*n* = 8 per group) using a random number generator: Control, DSS, C-1 and cC-1. The Control and DSS groups received daily oral gavage of 100 μL sterile PBS (pH 7.4) throughout the experiment. The C-1 and cC-1 groups were administered 100 μL suspensions containing 10⁹ CFU of C-1 or cC-1, respectively, in sterile PBS. Oral gavage was performed using a size 8 gavage needle attached to a 1 mL sterile syringe. Mice received probiotic intervention for 7 days prior to DSS exposure and continued throughout the DSS treatment period (prophylactic + therapeutic design). While the Control group received normal drinking water for the entire study. DSS, C-1, and cC-1 groups were provided normal water during day 1–7, followed by 2.5% dextran sulfate sodium (DSS, molecular weight 36,000-50,000 Da; MP Biomedicals, 160110, USA) solution from day 8–14. Throughout the experiment, the activity, behavior, and health of animals were observed daily, and the body weights were recorded. After 14 days of treatment, mice were sacrificed by cervical dislocation after the animals were anesthetized with 3:97 isoflurane and oxygen mix and tissues were excised and fixed in 4% paraformaldehyde or Carnoy’s fixative for histopathology analysis. The fecal samples were collected for gut microbiota analysis and untargeted metabolomic analysis; intestinal tissues were collected for transcriptome analysis, ELISA, Q-RT-PCR and Western Blotting.

### Evaluation of UC by disease activity index (DAI) score

During the DSS-induced ulcerative colitis experiment, daily measurements of body weight were recorded, and fecal characteristics were monitored^48^. Fecal occult blood was assessed using a commercial test kit (Mouse fecal occult blood test kit, Shanghai Enzyme-linked Biotechnology Co., Ltd., ml095013, China), with the DAI calculated as the composite score of weight loss, stool consistency, and fecal bleeding, as detailed in Table S1.

### Inflammatory cytokines expression in colon tissues by ELISA

Colon tissue samples were homogenized in pre-cooled PBS at a 1:9 (w/v) ratio using a tissue homogenizer (Shanghai Jingxin, China). The resulting homogenate was subjected to ELISA using specific kits for TNF-*α* (Absin, abs552204, China), IL-1*β* (Absin, abs552801, China), IL-6 (Absin, abs552805, Shanghai, China), and IFN-*γ* (Elabscience Biotechnology Co., Ltd, E-EL-M0048, China) following manufacturer’s instructions to quantify TNF-*α*, IL-1*β*, IL-6 and IFN-*γ* levels in colonic tissues.

### Western blotting

Colon tissues were lysed in RIPA buffer (Epizyme Biotechnology Co., Ltd, PC101, China) containing protease inhibitor cocktail (Epizyme Biotechnology Co., Ltd, GRF103, China) and followed by centrifugation at 10,000 × *g* for 20 min at 4 °C. The supernatant was collected and protein concentration determined using BCA assay (Epizyme Biotechnology Co., Ltd, ZJ102, China). Equal amounts of protein (30 μg per lane) were denatured in 5×sample buffer (Epizyme Biotechnology Co., Ltd, LT101S, China) at 95 °C for 5 min, resolved on SDS-PAGE gels. SDS-page gel was prepared using One-Step PAGE Gradient Gel Fast Reserve Kit (Vazyme Biotech Co. Ltd, E306-01, China). Samples were transferred to 0.2 μm PVDF membranes (Millipore, ISEQ00010, USA) at 300 mA for 90 min at 4 °C. Membranes were blocked with 5% non-fat milk in TBST for 1 h at room temperature. Individual proteins were detected using 1:1000 dilution of primary antibodies, as follows: Occludin (1:1000, CST, 91131), Claudin-1 (1:1000, Abcam, ab307692), and GAPDH (1:2000, Boster Biological Technology, BM3874, China). After washing three times with TBST (10 min each), membranes were incubated with horseradish peroxidase-conjugated secondary antibodies (1:5000, Boster Biological Technology, BA1054, China) for 2 h at room temperature. Following three additional washes with TBST (10 min each), protein bands were visualized using enhanced chemiluminescence substrate (Vazyme Biotech Co. Ltd, E433, China) and a chemiluminescence imaging system (Servicebio, China), with densities quantified using ImageJ and normalized to GAPDH.

### Histopathology analysis of colon tissues

Following euthanasia, colon tissue samples were rinsed with pre-cooled PBS and fixed in either 4% paraformaldehyde or Carnoy’ s fixative. Paraffin-embedded sections (4 μm thick) were prepared from PFA-fixed tissues and sequentially stained with hematoxylin and eosin (H&E). Dehydration was performed using anhydrous ethanol and xylene, followed by mounting with neutral balsam for histological evaluation. Carnoy’ s-fixed specimens underwent sequential staining with alcian blue, Schiff’s reagent, hematoxylin, and Scott’ s tap water substitute (AB-PAS staining), followed by identical dehydration and mounting procedures. Digitization of H&E and AB-PAS-stained sections was conducted using a Pannoramic DESK scanner (3DHISTECH, Hungary). Histological damage was assessed blindly by three independent pathologists using a scoring system as detailed in Table S2^49^.

### Gut microbiota by 16S rRNA sequencing and data analysis

Gut microbiota analysis followed our previously established protocol^31^. Total genomic DNA was extracted from fecal samples using FastPure Stool DNA Isolation Kit (Vazyme Biotech Co. Ltd, DC501, China), and the V3-V4 hypervariable regions of bacterial 16S rRNA genes were amplified via PCR using universal primers 338 F (5’-ACTCCTACGGGAGGCAGCAG-3’) and 806 R (5’-GGACTACHVGGGTWTCTAAT-3’) synthesized by Sangon Biotech (Shanghai, China). The PCR reaction mixture included 4 μL 5 × Fast Pfu buffer, 2 μL 2.5 mM dNTPs, 0.8 μL each primer (5 μM), 0.4 μL Fast Pfu polymerase, 10 ng of template DNA, and ddH_2_O to a final volume of 20 µL. PCR amplification cycling conditions were as follows: initial denaturation at 95 °C for 3 min, followed by 27 cycles of denaturing at 95 °C for 30 s, annealing at 55 °C for 30 s and extension at 72 °C for 45 s, and a single extension at 72 °C for 10 min, and end at 4 °C. The PCR product was detected, purified and quantified using Qubit 4.0 (Thermo Fisher Scientific, USA). Purified amplicons were pooled in equimolar amounts and paired-end sequenced on an Illumina Nextseq2000 platform (Illumina, USA) according to the standard protocols by Majorbio Bio-Pharm (Shanghai, China). After demultiplexing, the resulting sequences were quality filtered with fastp v0.19.6 and merged with FLASH. Quantified amplicons were processed with the TruSeq™ DNA Sample Prep Kit (Illumina, USA) for library construction, followed by sequencing and bioinformatic analysis to characterize microbial community profiles. The DADA2 method in Qiime2 v1.91 was used for quality filtering, sequence joining, and chimera removal, resulting in the generation of amplicon sequence variants. Taxonomic assignments were achieved using the Silva v138 database. The sequenced data were submitted to SRA with the accession number of PRJNA1389841.

The microbiota composition of each sample was calculated at the phylum, class, order, family, and genus levels. The abundance of each species in samples was obtained using QIIME2.0. Alpha diversity was analyzed using indices of ACE and Chao1 for richness and Shannon and Simpson for diversity. Principal coordinate analysis (PCoA) was employed using both Bray-Curtis and weighted-Unifrac distances, with PERMANOVA being used to analyze the statistical significance between groups. To further characterize the microbial composition of the samples, k-means clustering was performed at the OTU level based on Bray-Curtis distance. The k-means clustering analysis of Bray-Curtis distances for core bacterial genera was performed using the vegan (v2.6–4), fpc (v2.2–10), and cluster (v2.1.4) packages in R (v4.5.0), with subsequent visualization of the results. The linear discriminant analysis effect size (LDA LEfSe) algorithm is a high-dimensional approach for discovering and explaining biomarkers. It identifies genomic features (genes, pathways, or taxa) that characterize differences between two or more biological conditions. It was performed using the LEfSe package in R (v 3.3.1) with the following parameters: Kruskal-Wallis test alpha = 0.05, LDA score logarithmic threshold = 3.0, and multi-class analysis strategy set to all-against-all.

### Untargeted metabolomics analysis of intestinal contents

Colonic intestinal contents were collated for untargeted metabolomics. A 400 μL solution (Methanol: Water = 7:3, V/V) was added to the 20 mg sample, vortexed and then centrifuged to remove the sediment. All samples were detected using positive ion and negative ion conditions in LC/MS. For each sample, one aliquot was analyzed using positive ion conditions and was eluted from T3 column (ACQUITY Premier HSST3, Waters) using 0.1% formic acid in water as solvent A and 0.1% formic acid in acetonitrile as solvent B in the following gradient: 5–20% in 2 min, increased to 60% in the following 3 min, increased to 99% in 1 min and held for 1.5 min, then come back to 5% mobile phase B within 0.1 min, held for 2.4 min. The analytical conditions were as follows: column temperature, 40 °C; flow rate, 0.4 mL/min; injection volume, 4 μL; Another aliquot was used under negative ion conditions, and was the same as the elution gradient of positive mode. The raw data were submitted to OMIX database at the China National Center for Bioinformation (CNCB) under accession number of OMIX016979.

Data acquisition was performed using information-dependent acquisition (IDA) mode on Analyst TF 1.7.1 (Sciex, Concord, ON, Canada). Raw mass spectrometry data were converted to mzXML format using ProteoWizard, followed by peak extraction, alignment, and retention time correction with the XCMS program. Metabolite identification was achieved by matching processed peaks against multiple databases, including Metware Biotechnology Co., Ltd.‘s (Wuhan, China) in-house library (>5,000 metabolite standards with validated MS/MS spectra), integrated public repositories [HMDB v5.0 (https://hmdb.ca) and KEGG (https://www.genome.jp/kegg/)], and predictive tools (MetDNA, http://metdna.zhulab.cn/). Annotations were assigned at Level 1 (confirmed by authentic standard) or Level 2 (putatively annotated by database match) according to Metabolomics Standards Initiative (MSI) guidelines. Isomer discrimination was based on retention time comparison with authentic standards when available, or MS/MS spectral matching of characteristic fragment ions. Only annotations with MS/MS spectral similarity scores >0.8 (in-house database) or >0.7 (public databases) were retained. To mitigate batch effects, samples were randomized prior to analysis, and pooled quality control (QC) samples were injected every 10 samples throughout the analytical run. Features with coefficient of variation (CV) > 30% in QC samples were excluded. Compounds were further filtered using composite identification scores ≥0.5 (integrated metric of spectral similarity and retention time accuracy), yielding the final curated dataset. The remaining compounds were merged between positive and negative ionization modes, retaining those with the highest qualitative confidence levels and minimal CV values to generate the final curated dataset. Unsupervised PCA was performed using the prcomp function in R (v4.5.0). For orthogonal partial least squares discriminant analysis (OPLS-DA), data were log₂-transformed and mean-centered. VIP values, score plots, and permutation plots were generated using the MetaboAnalystR package (v1.0.1). A permutation test (200 permutations) was performed to prevent overfitting.

### Transcriptome sequencing and analysis of intestinal tissues

Total RNA was extracted from colon tissue using TRIzol reagent (Invitrogen, 15596026, USA) and quantified with Qubit fluorometer (Thermo Fisher Scientific) and Qsep400 bioanalyzer (Guangding Biotech, China). mRNA was enriched by Oligo(dT) magnetic beads (Vazyme Biotech Co. Ltd, N401, China), followed by reverse transcription with random hexamer primers and second-strand cDNA synthesis with end repair and dA-tailing (Yeasen, 12415ES02, China) for library construction. Preliminary quantification of the libraries was performed using a Qubit fluorometer (Thermo Fisher Scientific, USA), followed by insert size assessment with a fragment analyzer (Guangding Biotech, China). Qualified libraries were proportionally pooled based on target sequencing volume. The cDNA libraries were sequenced on the Illumina sequencing platform by Metware Biotechnology Co., Ltd. (China). The sequenced data were submitted to SRA with the accession number of PRJNA1390322.

The raw data passed quality control and mapped to the reference genome (GCA_02937485.1), differential expression analysis of two groups was performed using the DESeq2 (1.38.3). The resulting *P* values were adjusted using the Benjamini and Hochberg’s approach for controlling the false discovery rate. Differentially expressed genes were identified using thresholds of |log₂ (Fold Change)| ≥ 1 and *P*_*adj*_ < 0.05. For the differentially expressed genes, the GO (Gene Ontology) and KEGG (Kyoto Encyclopedia of Genes and Genomes) enrichment were analyzed.

To validate the transcriptomic analysis of the mRNA sequencing data, Quantitative real-time PCR (Q-RT-PCR) was used to quantify gene expression levels from samples collected above, and primer sequences provided in Table S3. Total RNA was extracted from colon tissue samples using TRIzol reagent (Invitrogen, 15596026, USA). RNA concentration and purity was measured using a NanoDrop 2000 spectrophotometer (Thermo Fisher, USA), with A₂₆₀/A₂₈₀ ratios between 1.8-2.0 considered acceptable. Reverse transcription to cDNA was performed with HiScript IV All-in-One Ultra RT SuperMix (Vazyme Biotech Co. Ltd, R433, China). Q-RT-PCR was conducted using SupRealQ Ultra Hunter SYBR qPCR Master Mix (U + ) (Vazyme Biotech Co. Ltd, Q713, China), 0.25 µM of each specific primer set and 1 µl of cDNA on a LightCycler® 96 Instrument (Roche Life Science, Switzerland). The relative expression of gene was analyzed using the 2^-ΔΔCt^, with *GAPDH* as the internal reference.

### Statistical analysis

All in vitro experiments were performed with three independent biological replicates (*n* = 3), each with three technical replicates. Animal experiments included 8 mice per group (*n* = 8) for phenotypic assessments, with 3–6 per group randomly selected for subsequent molecular, microbiome, and metabolomic analyses as indicated in figure legends. The data were expressed as mean ± SD. SPSS software (v23.0) and GraphPad (v10.0) were used for statistical analysis. Statistical comparisons were assessed by student’s *t* test (for two-group comparisons) or one-way analysis (ANOVA), followed by Tukey’s HSD post-hoc test for multiple group comparisons, with *P* < 0.05 as statistical difference.

## Supplementary information

Supplementary information

## Data Availability

The raw data reported in this article were deposited in the NCBI Sequence Read Archive under theaccession number PRJNA1389841 and PRJNA1390322, and Additional data supporting the findings of thisstudy are available within the article and its Supplementary Information.
