# Supplementary material for: Enhanced gastrointestinal stability and therapeutic efficacy of biofilm self-coated Bacillus amyloliquefaciens C-1 probiotic in ulcerative colitis
Source: NPJ Sci Food. 2026 May 19;10:240. doi: 10.1038/s41538-026-00889-2 (PMC13454628; doi:10.1038/s41538-026-00889-2)
Supplement: Supplementary file 1 [file 41538_2026_889_MOESM1_ESM.docx]

**Supplementary materials**

**Table S1.** Disease activity index (DAI) score of the cC-1 treated UC mice.

**Table S2.** Histological scoring system.

**Table S3.** Primers used for Q-RT-PCR.

**Table S4.** The relative abundance of gut microbiota at the phylum level.

**Table S5.** The relative abundance of gut microbiota at the genus level.

**Table S6.** The composition of different categories of non-targeted metabolomics identifiers.

**Data S1.** Untargeted metabolomics metabolite identification information.

**Figure S1**. Evaluation of UC by DAI score of mice after 7 days intervention.

**Figure S2**. H&E staining of mice colon tissue.

**Figure S3.** AB-PAS staining of mice colon tissue.

**Figure S4.** Q-RT-PCR verification of the randomly selected genes expression. (A) The optimal number of clusters based on the gap statistic. (B) K-means clustering plot by Bray-Curtis distance based on the optimal number clusters. (C) Alpha diversity index. (D) PCoA on OTU level. (E) The relative abundance of gut microbiota at the phylum level. (F) The relative abundance of gut microbiota at the genus level. (G) LDA scores bar chart among two groups. ***P* < 0.01, ****P* < 0.001.

**Figure S5.** Original figures of western blotting for three repeats.

**Table S.1.** Disease activity index (DAI) score of the cC-1 treated UC mice

| **Score** | **Weight Loss（%）** | **Stool Consistency** | **Hematochezia** |
| --- | --- | --- | --- |
| 0 | None | Normal | Negative hemocult |
| 1 | 1-5 | Soft but still formed | Negative hemocult |
| 2 | 6-10 | Soft | Positive hemocult |
| 3 | 11-18 | Very soft; wet | Blood traces in stool visible |
| 4 | >18 | Watery diarrhea | Gross rectal bleeding |

**Table S.2.** Histological scoring system.

| Inflammatory cell infiltrate | | Score 1 | Intestinal architecture | | Score 2 |
| --- | --- | --- | --- | --- | --- |
| Severity | Extent |  | Epithelial changes | Mucosal architecture |  |
| Mild | Mucosa | 1 | Focal erosions |  | 1 |
| Moderate | Mucosa and submucosa | 2 | Erosions | Focal ulceration | 2 |
| Marked | Transmural | 3 |  | Extended ulceration/ granulation tissue and pseudopolyps | 3 |
| scores 1+ scores 2 = 0-6 | | | | | |

**Table S.3.** Primers used for Q-RT-PCR.

| **Gene** |  | **Sequence (5’-3’)** |
| --- | --- | --- |
| *GAPDH* | Forward | AGGTCGGTGTGAACGGATTTG |
|  | Reverse | TGTAGACCATGTAGTTGAGGTCA |
| *Icam1* | Forward | GTGATGCTCAGGTATCCATCCA |
|  | Reverse | CACAGTTCTCAAAGCACAGCG |
| *Itgal* | Forward | CCAGACTTTTGCTACTGGGAC |
|  | Reverse | GCTTGTTCGGCAGTGATAGAG |
| *Itgb2* | Forward | TCCTGGGATCTGCTGTGTC |
|  | Reverse | AGTTCAGCTTCTGGCACC |
| *Rac2* | Forward | GACAGTAAGCCGGTGAACCTG |
|  | Reverse | CTGACTAGCGAGAAGCAGATG |
| *Vav1* | Forward | CTTAACAACCTGCTTCCCCAG |
|  | Reverse | CAGGGTGTAGATGACCTTTCC |

**Table S.4.** The relative abundance of gut microbiota at the phylum level.

| **Phylum** | **Control** | **DSS** | **cC-1** |
| --- | --- | --- | --- |
| Bacteroidota | 50.56 | 40.31 | 55.09 |
| Firmicutes | 40.76 | 43.39 | 27.88 |
| Patescibacteria | 3.62 | 9.39 | 11.24 |
| Campylobacterota | 2.48 | 3.80 | 0.61 |
| Actinobacteriota | 1.24 | 1.23 | 2.44 |
| Desulfobacterota | 1.15 | 1.10 | 1.75 |
| Proteobacteria | 0.08 | 0.58 | 0.88 |
| Deferribacterota | 0.01 | 0.13 | 0.01 |
| Cyanobacteria | 0.03 | 0.02 | 0.04 |
| Verrucomicrobiota | 0 | 0.01 | < 0.01 |
| Others | 0.08 | 0.04 | 0.06 |

**Table S.5.** The relative abundance of gut microbiota at the genus level.

| **Genus** | **Control** | **DSS** | **cC-1** |
| --- | --- | --- | --- |
| norank_f__Muribaculaceae | 32.72 | 24.26 | 44.99 |
| Lactobacillus | 23.27 | 8.83 | 9.93 |
| Candidatus_Saccharimonas | 3.62 | 9.39 | 11.24 |
| norank_o__Clostridia_UCG-014 | 4.37 | 13.09 | 4.53 |
| Prevotellaceae_UCG-001 | 2.04 | 9.39 | 1.08 |
| Lachnospiraceae_NK4A136_group | 2.11 | 5.77 | 1.64 |
| Bacteroides | 4.95 | 0.66 | 2.24 |
| Helicobacter | 2.48 | 3.80 | 0.61 |
| Odoribacter | 1.89 | 2.28 | 1.83 |
| Alistipes | 4.51 | 0.72 | 0.68 |
| Others | 18.03 | 21.80 | 21.25 |

**Table S.6.** The composition of different categories of non-targeted metabolomics identifiers.

| **Category** | **Proportion** | **Category** | **Proportion** |
| --- | --- | --- | --- |
| Alcohol and amines | 4.95 | Hormones and it related compounds | 4.47 |
| Aldehyde, Ketones, Esters | 8.02 | Lignans and Coumarins | 0.46 |
| Alkaloids | 1.38 | Nucleotide and Its metabolites | 2.06 |
| Amino acid and Its metabolites | 23.04 | Organic acid and Its derivatives | 9.05 |
| Benzene and substituted derivatives | 14.69 | Phenolic acids | 0.17 |
| Bile acids | 0.71 | Quinones | 0.01 |
| Carbohydrates and Its metabolites | 1.35 | SP | 0.35 |
| Coenzyme and vitamins | 0.55 | Steroids | 0.65 |
| FA | 4.70 | Tannins | 0.06 |
| Flavonoids | 0.97 | Terpenoids | 2.03 |
| GL | 0.77 | Tryptamines, Cholines, Pigments | 0.40 |
| GP | 2.36 | Others | 5.45 |
| Heterocyclic compounds | 11.35 |  |  |

**Data S1. Untargeted metabolomics metabolite identification information.**

*Note: This file is provided as a separate Excel spreadsheet due to its large size.*

**Figure S1. Evaluation of UC by DAI score of mice after 7 days intervention.**

**Figure S2. H&E staining of mice colon tissue.**

**Figure S3.** **AB-PAS staining of mice colon tissue.**

**Figure S4.** Q-RT-PCR verification of the randomly selected genes expression. (A) The optimal number of clusters based on the gap statistic. (B) K-means clustering plot by Bray-Curtis distance based on the optimal number clusters. (C) Alpha diversity index. (D) PCoA on OTU level. (E) The relative abundance of gut microbiota at the phylum level. (F) The relative abundance of gut microbiota at the genus level. (G) LDA scores bar chart among two groups. ***P* < 0.01, ****P* < 0.001.

**Figure** S5. Original figures of western blotting for three repeats.
